# Pilot study: Unveiling the impact of bisphenol A and phthalate exposure on women with asthma

**DOI:** 10.1097/MD.0000000000039840

**Published:** 2024-09-27

**Authors:** Kyung-Min Ahn, Min-Suk Yang, Ha-Kyeong Won, Jung Ah Lim

**Affiliations:** a Department of Internal Medicine, Ewha Womans University College of Medicine, Seoul, Korea; b Division of Allergy and Clinical Immunology, Department of Internal Medicine, Seoul National University College of Medicine, Seoul, Korea; c Department of Internal Medicine, SMG-SNU Boramae Medical Center, Seoul, Korea; d Department of Internal Medicine, Veterans Health Service Medical Center, Seoul, Korea; e Division of Endocrinology and Metabolism, Department of Internal Medicine, National Medical Center, Seoul, Korea.

**Keywords:** asthma, phthalates

## Abstract

Endocrine disruptors are considered estrogenic disruptors, and recent researches suggested that they may have a link to the severity of asthma. We aim to validate the correlation between endocrine disruptors and various clinical measurements of asthma, depending on the menopausal status. A pilot case–control study was performed in female asthmatic patients who visited allergy clinic in SMG-SNU Boramae Medical Center. Medical information and the urinary concentrations of 4 endocrine disruptors on their first visit were collected and analyzed: bisphenol A, mono (2-ethyl-5-hydroxyhexyl) phthalate, mono (2-ethyl-5-oxohexyl) phthalate, and mono-n-butyl phthalate. A total of 35 female participants enrolled in the study, including 20 asthmatic patients and 15 healthy controls. The average concentrations of urinary endocrine disruptors in patient and control group did not demonstrate significant differences. Twenty asthmatic patients were divided into 2 groups according to their menstrual state. Using the Spearman rank correlation test in premenopausal asthmatic patients (n = 7), we found negative correlations between urinary concentration of mono-n-butyl phthalate and asthma control test score, as well as postbronchodilator forced expiratory flow at 25% to 75% of forced vital capacity (*P*-value = .007 and .04, respectively). In contrast, it did not show any correlation with asthma control test or postbronchodilator forced expiratory flow at 25% to 75% of forced vital capacity (*P*-value = 1.00 and .74, respectively) in postmenopausal group (n = 13). Endocrine disruptors might have an impact on the decline of small airway function and asthma management among premenopausal, but not postmenopausal, female asthmatic patients.

## 1. Introduction

Asthma is a chronic respiratory inflammatory disorder, characterized by episodic airway hyperresponsiveness and recurrent airway obstruction. This airway disease leads to various symptoms in a wide range of severity with an increasing prevalence worldwide. According to the 2021 Global Asthma Network (GAN) Phase I study, the prevalence of current asthma symptoms was 9.1% among children, 11.0% among adolescents, and 6.6% among adults.^[1,2]^ In Korean National Health and Nutrition Examination Survey databases, the estimated prevalence rate of asthma was 2.3% in adults and 4.1% in the elderly, which has steadily increased in last decade.^[3]^ Although there have been continuous attempts for effective treatments, more than 10% of asthmatic patients do not achieve sustained asthma control.^[4]^ This results in heavy social and economic burdens globally in both pediatric and adult patients.^[5,6]^ On a global scale, asthma is positioned as the 24th leading cause of Years Lived with Disability (YLDs) and the 34th in terms of Disability-Adjusted Life Years (DALYs). Asthma emerges as a substantial contributor to the worldwide economic burden, encapsulating both direct and indirect costs.^[1,7]^

Over recent years, invention of plastic has brought great benefits to human society in last century. As important chemical building blocks in manufactory, bisphenol A (BPA) and phthalates have become the most widely used plasticizers in producing, packaging, or delivering.^[8]^ Despite its practicality, the impact of plastics on human body is not neglectable.^[9]^ The production and use of plastic and its derivatives, particularly BPA and phthalates, have risen dramatically which increased concerns of their negative influence on the development of a number of disorders, including metabolic, allergic, and immunologic dysfunctions.^[10]^ These highest volume chemicals would interact with estrogen and androgen receptors^[11]^ and have been measured in human fluids and tissues, including blood, placental tissue, amniotic fluid, breast milk, urine, semen, and follicular fluid.^[12]^ Previous studies have reported the possible impact of plastics as endocrine disrupting chemical substances in vivo and in vitro.^[13–15]^ Since they are ubiquitously present in modern daily life, their possible immunomodulatory effects in long-term exposure cause concerns to patients with chronic pulmonary diseases, such as asthma.^[15,16]^ Although previous studies have demonstrated the possible relation between endocrine disruptors and respiratory conditions on neonatal and children, their link on adult asthmatic patients are still in need to be verified.^[17,18]^ Moreover, there has not been prospective analysis on relationship between the levels of endocrine disruptors in body and asthma in Korea. In this pilot study, we aim to validate the influence of endocrine disruptors on asthma and the correlation between environmental disruptors and various clinical measurements of asthma, depending on the menopausal status.

## 2. Materials and methods

### 2.1. Study population

Female asthma patients who visited allergy clinic in Seoul Metropolitan Government - Seoul National University (SMG-SNU) Boramae Medical Center and agreed to participate in this study were prospectively recruited. Asthma was diagnosed when one or more of the following criteria were satisfied: (1) an improvement in forced expiratory volume in 1 second (FEV1) of at least 12% and at least 200 mL, 15 to 20 minutes after administration of an inhaled rapid-acting β2-agonist; (2) a decreased FEV1 value by more than 15% compared with the baseline FEV1 after the mannitol bronchial provocation test, or (3) an improvement in FEV1 of at least 20% and at least 200 mL after 2 weeks of treatment with an anti-inflammatory agent such as an inhaled corticosteroid or a leukotriene receptor antagonist. Spirometry measurements included prebronchodilator (preBD) FEV1, postbronchodilator (postBD) FEV1, preBD forced vital capacity (FVC), postBD FVC, preBD FEV1/FVC, postBD FEV1/FVC, preBD forced expiratory flow at 25 to 75% of forced vital capacity (FEF25–75%), and postBD FEF25–75%.FEV1 and FVC were presented as percent of predicted value (%pred). Severe asthma exacerbation is defined as experience of either admission or emergency department visit due to asthma attack or systemic corticosteroid use during past 1 year.^[19]^ Medical information and survey questionnaire was collected including age, body mass index (BMI), state of menstruation, asthma duration and severity, the presence of comorbidities such as allergic rhinitis, atopic dermatitis or chronic rhinosinusitis, asthma control test (ACT) score, Quality of Life Questionnaire for Adult Korean Asthmatics (QLQAKA),^[20]^ results of spirometry, eosinophil count, and total IgE level between January and December 2014. Participants who receive contraceptive drugs or equipment which contains progestin or estrogen derivatives were excluded. Asthmatic patients who have destructive lung disease on the chest X-ray examination such as a tuberculous destroyed lung and lung cancer were also excluded.

The control group were female volunteers who agreed to participate in this study and had no medical history of allergic diseases. Their medical information was also collected.

### 2.2. Study design and measurements

This study was designed as a case–control study. The participants’ urinary endocrine disruptor levels were measured after the first visit of allergy clinic. All participants collected a random urine sample. In order to yield the accurate concentrations of endocrine disruptors, patients required to avoid any allergy medications including systemic corticosteroids, inhaled corticosteroids, long acting β2 agonists, methylxanthine derivative agents, leukotriene controllers, and antihistamines for at least 7 days in advance of the measurement of urinary endocrine disruptors. All urine samples were collected and stored at −20 degrees Celsius until the analysis. Four types of the urinary endocrine disruptors were measured: BPA, mono (2-ethyl-5-hydroxyhexyl) phthalate (MEHHP), mono (2-ethyl-5-oxohexyl) phthalate (MEOHP), and mono-n-butyl phthalate (MnBP). Each concentration of the urinary endocrine disruptor was adjusted by urinary creatinine level of the participant and expressed as µg of urinary endocrine disruptors over g of urinary creatinine (µg/g).

The primary outcome was the comparisons of urinary concentrations of endocrine disruptors between asthmatic patients and non-asthmatic individuals. The secondary outcome was the investigation on the correlations between urinary concentrations of endocrine disruptors and factors associated with airway inflammation, hypersensitivity, severity of disease in asthmatic patients, depending on their menopausal state.

### 2.3. Statistical analysis

All analyses were performed using SPSS statistical software (version 22.0; SPSS Inc., Chicago, IL). Two-sided *P*-values < .05 were considered statistically significant. Mann–Whitney test was used to evaluate the continuous variables and Spearman correlation was used to analyze the categorical variables between groups. Variables were presented using the number of valid cases (N), means ± standard deviations, or medians (ranges).

This study was approved by the Institutional Review Board of SMG-SNU Boramae Medical Center, and informed consent was obtained from all participants (IRB No. 16-2014-22). Human rights were respected in accordance with the Helsinki Declaration.

## 3. Results

### 3.1. Baseline characteristics and urinary concentration of endocrine disruptors in study subjects

A total of 35 female participants enrolled in the study, including 20 asthmatic patients and 15 healthy controls. The mean age of patients was 53.8 ± 19.8 years and average concentrations of urinary endocrine disruptors were as follows: 3.66 ± 7.99 µg/g for BPA, 27.77 ± 17.91 µg/g for MEHHP, 15.80 ± 11.71 µg/g for MEOHP, and 38.95 ± 26.23 µg/g for MnBP, respectively. Spirometry was performed in each of 20 female asthmatic patients. The average preBD FEV1 and FVC were 92.6 ± 22.8% and 92.7 ± 16.8% and postBD FEV1 and FVC were 95.6 ± 21.5% and 94.2 ± 16.3%, respectively. Pre and postBD FEV1/FVC were 81.6 ± 24.2% and 76.8 ± 10.4% and pre and postBD FEF25–75% were 71.8 ± 40.4% and 77.7 ± 41.0%, respectively.

The mean age of 15 health female controls was 46.3 ± 14.2 years and average concentrations of urinary endocrine disruptors were 2.87 ± 2.39 µg/g for BPA, 43.01 ± 59.68 µg/g for MEHHP, 23.57 ± 35.33 µg/g for MEOHP, and 40.63 ± 24.85 µg/g for MnBP, respectively. There was no significant difference in baseline characteristics of asthmatic patients and healthy controls, including age and BMI. Two groups did not demonstrate significant difference in concentrations of urinary BPA, MEHHP, MEOHP, or MnBP (*P* = 0. 71, 0.29, 0.36, and 0.85, respectively). There was no statistically significant difference observed in smoking prevalence between the control and case groups (*P* = .17).

### 3.2. Baseline characteristics of asthmatic patients according to menopausal state

Twenty asthmatic patients were divided into 2 groups according to their menstrual state: premenopausal (n = 7) and postmenopausal (n = 13) (Table 1). The baseline characteristics did not show significant difference between 2 groups, except for their ages. The average ages of premenopausal and postmenopausal groups were 34.6 ± 16.0 and 64.1 ± 12.7 years (*P* < .001). The difference between BMI (premenopausal female patients 22.6 ± 4.2 and postmenopausal female patients 24.6 ± 5.2, *P* = .42), asthma duration (4.3 ± 5.4 and 10.5 ± 8.3, years, *P* = .10), 1 (14.3%) premenopausal and 4 (30.8%) postmenopausal patients experienced severe asthma exacerbation in the past 12 months according to (*P* = .79), ACT score (16.7 ± 2.3 and 16.9 ± 4.3, *P* = .89), and QLQAKA (64.0 ± 10.7 and 59.2 ± 14.0, *P* = .17) did not show significant differences between 2 groups.

**Table 1:** Baseline characteristics of the study subjects.

	Premenopausal (n = 7)	Postmenopausal (n = 13)	*P*-value
Age	34.6 ± 16.0	64.1 ± 12.7	<.001
Height	159.4 ± 3.7	153.7 ± 6.7	.05
Body weight	57.7 ± 11.8	58.2 ± 13.0	.94
BMI	22.6 ± 4.2	24.6 ± 5.2	.42
Asthma duration	4.3 ± 5.4	10.5 ± 8.3	.10
Severe asthma exacerbation in the past 12 months (%)*	1 (14.3)	4 (30.8)	.79
Asthma control test	16.7 ± 2.3	16.9 ± 4.3	.89
QLQAKA	64.0 ± 10.7	59.2 ± 14.0	.17
Allergic rhinitis (%)	4 (57.1)	8 (61.5)	1.00
Atopic dermatitis (%)	2 (28.6)	2 (15.4)	.91
Chronic rhinosinusitis (%)	0 (0)	3 (23.1)	.47
preBD FEV1 (%pred)	92.0 ± 12.2	92.9 ± 27.3	.93
postBD FEV1 (%pred)	94.3 ± 11.9	96.2 ± 25.6	.85
preBD FVC (%pred)	96.3 ± 13.1	90.8 ± 18.6	.50
postBD FVC (%pred)	96.3 ± 14.3	93.0 ± 17.7	.68
preBD FEV1/FVC (%)	80.1 ± 16.4	82.3 ± 28.1	.86
postBD FEV1/FVC (%)	81.1 ± 9.5	74.5 ± 10.5	.18
preBD FEF25–75% (%pred)	73.8 ± 35.1	71.0 ± 43.6	.90
postBD FEF25–75% (%pred)	83.8 ± 33.8	75.1 ± 44.8	.70
Eosinophil count (/mm^3^)	432.4 ± 295.5	301.9 ± 277.6	.39
Total IgE (IU/mL)	146.0 ± 167.4	84.8 ± 101.8	.41
BPA/creatinine (µg/g)	1.34 ± 0.91	4.92 ± 9.79	.35
MEHHP/creatinine (µg/g)	17.93 ± 11.48	33.06 ± 18.84	.07
MEOHP/creatinine (µg/g)	8.94 ± 7.27	19.50 ± 12.18	.05
MnBP/creatinine (µg/g)	24.01 ± 10.36	46.99 ± 28.90	.06

BMI = body mass index, BPA = bisphenol, MEHHP = mono (2-ethyl-5-hydroxyhexyl) phthalate, MEOHP = mono (2-ethyl-5-oxohexyl) phthalate, MnBP = mono-n-butyl phthalate, postBD = postbronchodilator, preBD = prebronchodilator, QLQAKA = Quality of Life Questionnaire for Adult Korean Asthmatics.

* Defined as experienced either of admission or emergency department visit due to asthma or systemic corticosteroid use during past 1 year.

The eosinophil count was 432.4 ± 295.5 and 301.9 ± 277.6 (*P* = .39) and total IgE were 146.0 ± 167.4 and 84.8 ± 101.8 (*P* = .41) in premenopausal and postmenopausal females, respectively. The results of spirometry were as below: preBD FEV1 (92.0 ± 12.2% and 92.9 ± 27.3% in premenopausal and postmenopausal female patients, respectively, *P* = .93), postBD FEV1 (94.3 ± 11.9% and 96.2 ± 25.6%, *P* = .85), preBD FVC (96.3 ± 13.1% and 90.8 ± 18.6%, *P* = .50), postBD FVC (96.3 ± 14.3 and 93.0 ± 17.7, *P* = .68), preBD FEV1/FVC (80.1 ± 16.4% and 82.3 ± 28.1%, *P* = .86), postBD FEV1/FVC (81.1 ± 9.5% and 74.5 ± 10.5%, *P* = .18), preBD FEF 25–75 (73.8 ± 35.1% and 71.0 ± 43.6%, *P* = .90), and postBD FEF25–75 (83.8 ± 33.8% and 75.1 ± 44.8%, *P* = .70).

The comparisons of average concentrations of endocrine disruptors between 2 groups did not show significant differences: BPA (1.34 ± 0.91 µg/g and 4.92 ± 9.79 µg/g in premenopausal and postmenopausal female patients, respectively, *P* = .35), MEHHP (17.93 ± 11.48 µg/g and 33.06 ± 18.84 µg/g, *P* = .07), MEOHP (8.94 ± 7.27 µg/g and 19.50 ± 12.18 µg/g, *P* = .05), and MnBP (24.01 ± 10.36 µg/g and 46.99 ± 28.90 µg/g, *P* = .06) (Table 1).

### 3.3. Outcomes of Spearman rank correlation test between environmental hormones and ACT and lung function test according to menopausal state

**Figure 1.**
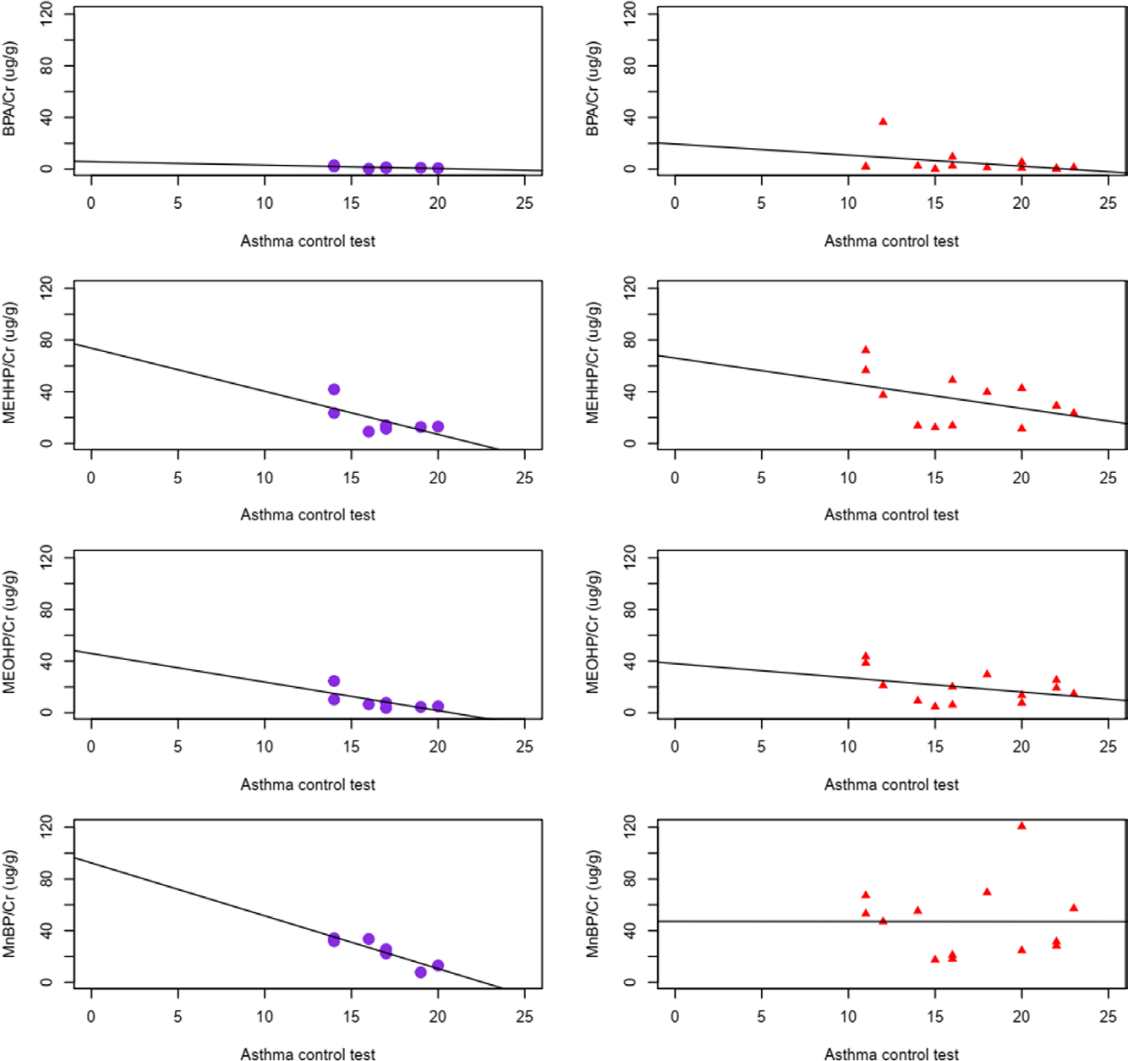
Correlation between endocrine disruptors and ACT score in asthma patients, premenopausal women (circle) and postmenopausal women (triangle).

**Figure 2.**
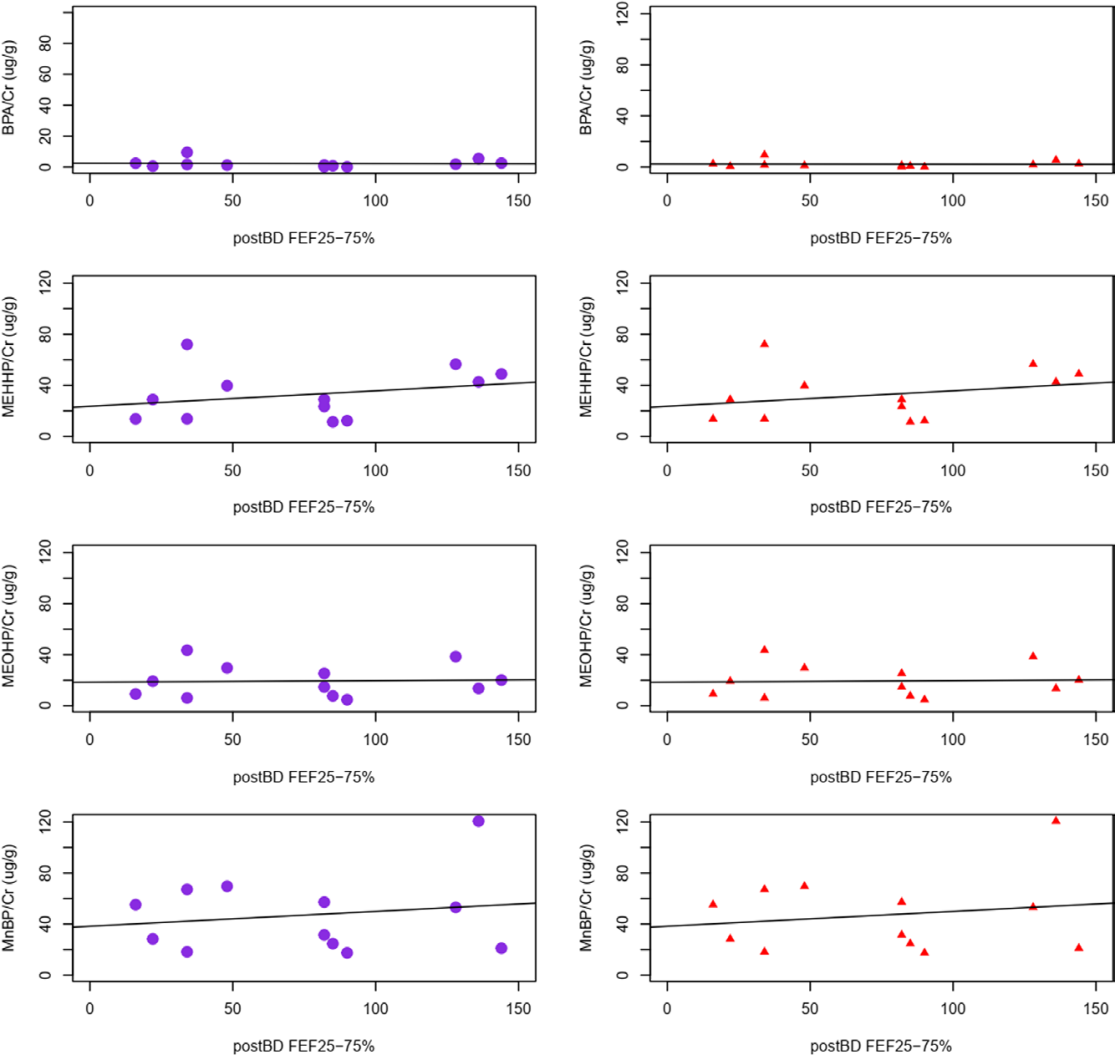
Correlation between endocrine disruptors and postBD FEF25–75% in asthma patients, premenopausal women (circle), and postmenopausal women (triangle).

Using the Spearman rank correlation test in the premenopausal asthmatic patients, we found negative correlations between urinary concentration of MnBP and ACT score, as well as postBD FEF25–75% (*P*-value = .007 and .04, respectively) (Tables 2 and 3, Figs. 1 and 2). The values of pre/postBD FEV1, pre/postBD FVC, pre/postBD FEV1/FVC ratio, eosinophil count, or total IgE levels did not show any correlation with endocrine disruptors in this group. Thus, endocrine disruptors showed influence on small airway inflammation and ACT score in premenopausal women. In contrast, the urinary concentration of MnBP of the postmenopausal group did not show any correlation with ACT or postBD FEF25–75% (*P*-value = 1.00 and .74, respectively).

**Table 2:** The correlations between endocrine disruptors and clinical measurements of premenopausal asthma patients.

	BPA/Cr (µg/g)		MEHHP/Cr (µg/g)		MEOHP/Cr (µg/g)		MnBP/Cr (µg/g)	
	ρ	*P*-value	ρ	*P*-value	ρ	*P*-value	ρ	*P*-value
Asthma control test	−0.56	.19	−0.44	.33	−0.75	.054	−0.89	**.007**
QLQAKA	−0.54	.22	0.14	.76	0.32	.48	0.07	.88
preBD FEV1 (%pred)	0.67	.10	0.18	.70	−0.23	.61	−0.31	.50
postBD FEV1 (%pred)	0.71	.07	0.29	.54	−0.14	.76	−0.18	.70
preBD FVC (%pred)	0.22	.64	0.20	.67	−0.29	.53	−0.31	.50
postBD FVC (%pred)	0.64	.12	0.36	.43	−0.07	.88	0.07	.88
preBD FEV1/FVC (%)	−0.43	.34	−0.04	.94	0.11	.82	-0.18	.70
postBD FEV1/FVC (%)	−0.52	.23	−0.34	.45	−0.40	.38	−0.70	.08
preBD FEF25–75% (%pred)	−0.50	.39	−0.30	.62	−0.30	.62	−0.70	.19
postBD FEF25–75% (%pred)	−0.80	.10	−0.40	.51	−0.40	.51	−0.90	**.04**
Eosinophil count	−0.43	.40	0.43	.40	0.43	.40	0.09	.87
Total IgE	0.30	.62	0.30	.62	0.30	.62	0.30	.62

Values that demonstrate significant correlations between parameters of asthma and endocrine disruptor are marked in bold.

QLQAKA = Quality of Life Questionnaire for Adult Korean Asthmatics, preBD = prebronchodilator, postBD = postbronchodilator.

**Table 3:** The correlations between endocrine disruptors and clinical measurements of postmenopausal asthma patients.

	BPA/Cr (µg/g)		MEHHP/Cr (µg/g)		MEOHP/Cr (µg/g)		MnBP/Cr (µg/g)	
	ρ	*P*-value	ρ	*P*-value	ρ	*P*-value	ρ	*P*-value
Asthma control test	−0.41	.17	−0.36	.23	−0.27	.37	0.00	1.00
QLQAKA	−0.57	.04	−0.01	.99	0.11	.73	0.09	.78
preBD FEV1 (%pred)	0.06	.86	0.24	.43	0.04	.89	−0.08	.79
postBD FEV1 (%pred)	0.04	.89	0.13	.67	−0.08	.79	−0.18	.55
preBD FVC (%pred)	0.14	.64	−0.07	.82	−0.31	.30	−0.37	.21
postBD FVC (%pred)	−0.02	.94	−0.05	.87	−0.23	.45	−0.26	.39
preBD FEV1/FVC (%)	−0.22	.48	0.21	.49	0.17	.58	0.33	.27
postBD FEV1/FVC (%)	0.22	.48	0.53	.06	0.29	.34	0.15	.62
preBD FEF25–75% (%pred)	0.17	.59	0.35	.24	0.07	.82	−0.07	.83
postBD FEF25–75% (%pred)	0.10	.77	0.26	.42	0.01	.97	−0.11	.74
Eosinophil count	−0.19	.60	−0.36	.31	−0.37	.29	−0.29	.43
Total IgE	−0.07	.87	−0.12	.77	0.00	1.00	0.03	.93

QLQAKA = Quality of Life Questionnaire for Adult Korean Asthmatics, preBD = prebronchodilator, postBD = postbronchodilator.

## 4. Discussion

In this pilot study, we found possible correlation between endocrine disruptors, particularly MnBP, and small airway dysfunction, and postBD FEF25–75%, and ACT score in female asthmatic patients, depending on the menopausal status. This indicates the potential role of endocrine disruptors in asthma severity. Previous epidemiological researches have reported the emerging cautions of BPA and phthalates, which are the most common environmental endocrine disruptors, that their exposure on neonatal and children during pregnancy or childhood is related to the development of asthma.^[17,18,21,22]^ However, most data involves children and adolescent patients, which focus their effect on the progress of asthma in the early stage of life,^[23,24]^ while there still is a controversy about its role in the adult asthmatic patients.^[25,26]^ In a number of prior studies, higher urinary BPA concentrations were associated with chronic diseases such as cardiovascular, diabetes and blood marks of inflammation and homeostasis, suggesting the role of endocrine disruptors in other systemic inflammatory diseases.^[27]^ Nevertheless, the elucidation of its detailed mechanism is still in need. In this study, we first aimed to investigate the impact of the endocrine disruptors on asthma in adult patients. However, correlation between urinary concentrations of endocrine disruptors and presence of asthma could not be substantiated. In order to examine their influence on airway inflammation, various clinical measurements of asthma were compared between premenopausal and menopausal female patients.

There has been current controversy between the correlation between endocrine disruptors and asthma. In the earlier study of urinary BPA concentration in asthmatic children, BPA concentration was significantly higher in patients than in control group (*P* = .001).^[28]^ The multiple logistic regression analysis also showed the importance of higher levels of BPA as a more significant predictor than passive smoking (*P* = .006 and .049, respectively), indicating the potential risk of BPA exposure in the development of bronchial asthma. This is in line with another study, in which higher levels of urinary BPA level were associated with decrease in small airway and lung function (FEV1/FVC) in a cross-sectional study of children and adolescent participants.^[10]^ Similar pattern was observed in adult population where mono benzyl phthalate levels showed association with asthma.^[29]^ On the other hand, positive associations between urinary phthalate and its metabolite levels and asthma were absent in Denmark study of preschool children, which appear to contradict previous studies. Several in vitro studies suggested the potential role of endocrine disruptor in T2 inflammation.^[30]^ BPA can activate pro-allergic T helper chemokines and cytokines, including CC chemokine ligand-1, IL-10, IL-5, and IL-13.^[15]^ Phthalate also induces the production of pro-allergic T2 cytokines including IL-4 and TNF-α in human macrophages.^[31]^ Our findings showed the correlations between urinary concentration of phthalate and small airway and ACT score in premenopausal asthmatic patients. These previous immunological findings would be one of the explanations for our findings, although further study should elucidate the mechanisms in molecular level.

The comparison of impacts of endocrine disruptors on female asthma patients according to their menopausal state was initiated based on the notion that there was solid evidence about the role of endocrine disruptors in interference of human sex hormones, including estrogen which is another trigger factor of T2 inflammations. G protein-coupled estrogen receptor 1 is a functional estrogen receptor involved in several organ systems, including reproductive, metabolic, and immune pathways.^[32]^ Recent epidemiological data indicate the role of sex steroid hormones in various lung diseases, including asthma.^[33]^ Clinical reports show an increased incidence and severity of asthma in women, suggesting the possible impact of estrogen on airway inflammation.^[34–36]^ Previous studies also revealed its influence on lung function.^[36]^ FEV1 and FVC are lowest in per-ovulation period which is marked by high circulating concentrations of estradiol in female adult patients.^[37,38]^ Phthalates are considered estrogenic disruptors, and recent researches suggested that they may have a link to the severity of asthma.

This study is the first study of effect of endocrine disruptors in female adult with asthma, depending on their menopausal status in Korea. However, we should acknowledge some limitations. First, we could not observe significant differences in concentrations of urinary BPA, MEHHP, MEOHP, or MnBP between healthy control and asthmatic patients (*P* = . 71, .29, .36, and .85, respectively). Considering possible role of endocrine disruptors on the T2 inflammation, we expected significant difference in the urinary concentration of endocrine disruptors between healthy control and asthmatic patients. Further studies with higher number of participants are needed to compare differences between 2 groups. Second, the nature of voluntary enrollments limited us from a thorough comparison of baseline characteristics and correlations between endocrine disruptors and asthmatic measurement in a great number of participants. Third, there are potential differences in sensitivity between urine and blood samples from an analytical perspective. Based on this brief and small-scale pilot study, we hope to conduct further studies with larger populations and subjects of different genders, with the aim of elucidating gender differences. Lastly, a short duration of study restricted an along-term monitoring the fluctuation of urinary concentrations in endocrine disruptors, which can theoretically influence their average values. A longitudinal study will permit us more insights of correlation between variability of these concentration and asthma in the future.

## 5. Conclusion

Endocrine disruptors might have an impact on small airway dysfunction and ACT score in premenopausal asthmatic patients, suggesting a potential role of endocrine disruptors in T2 inflammation.

## Author contributions

**Conceptualization:** Kyung-Min Ahn, Min-Suk Yang, Ha-Kyeong Won, Jungah Lim.

**Data curation:** Min-Suk Yang, Ha-Kyeong Won.

**Formal analysis:** Kyung-Min Ahn, Min-Suk Yang.

**Investigation:** Kyung-Min Ahn, Ha-Kyeong Won, Jungah Lim.

**Methodology:** Kyung-Min Ahn, Ha-Kyeong Won.

**Supervision:** Min-Suk Yang, Jungah Lim.

**Validation:** Jungah Lim.

**Visualization:** Min-Suk Yang.

**Writing – original draft:** Kyung-Min Ahn.

**Writing – review & editing:** Kyung-Min Ahn, Min-Suk Yang, Jungah Lim.
